# The impact of key monitoring policy on the usage of policy-related drugs in Hubei Province, China

**DOI:** 10.3389/fphar.2023.1088723

**Published:** 2023-02-15

**Authors:** Xiaotong Wen, Yue Wang, Xiaoze Chen, Yuxin Liu, Zongfu Mao

**Affiliations:** ^1^ School of Public Health, Wuhan University, Wuhan, China; ^2^ Global Health Institute, Wuhan University, Wuhan, China; ^3^ Xi’an Jiao Tong Liverpool University, Suzhou, China; ^4^ Dong Fureng Institute of Economic and Social Development, Wuhan University, Wuhan, China

**Keywords:** key monitoring, adjuvant drug, drug stewardship policy, rational drug use, interrupted time series analysis

## Abstract

**Introduction:** This study evaluated quantitatively the impact of the first batch of the catalog of Key Monitoring and Rational Use Drugs (KMRUD) in Hubei Province on policy-related drug use and expenditures.

**Methods:** This study is aimed to provide a basis for the successful implementation of subsequent catalogs of KMRUD, which may promote the standardization of clinical application of related drugs and effectively reduce drug expenses of the patients. Data on the procurement records of policy-related drugs from January 2018 to June 2021 were obtained from the Drug Centralized Procurement Platform of the Public Resources Trading Center in Hubei Province. Interrupted time-series (ITS) analysis was used in this study.

**Results:** After the implementation of the first batch of the catalog of KMRUD, the consumption of policy-related drugs decreased by 83.29% in 2020. The spending on policy-related drugs decreased by 83.93% in 2020. The introduction of the first batch of the catalog of KMRUD was associated with a significant decrease in the spending on policy-related drugs in the level (*p =* 0.001). Before the implementation of the KMRUD catalog policy, the Defined Daily Doses (DDDs) (β_1_ = -32.26 *p* < 0.001) and spending (β_1_ = -3662.19 *p* < 0.001) on policy-related drugs showed a downward trend. In the aggregated ITS analysis, the Defined Daily Dose cost (DDDc) of policy-related drugs decreased significantly in the trend (*p* < 0.001). After the implementation of the KMRUD catalog policy, the monthly procurement volume of 10 policy-related drugs have a significant downward trend (*p* < 0.05), and 4 policy-related drugs have a significant upward trend (*p* < 0.05).

**Conclusion:** After the policy intervention, the total DDDc on policy-related drugs indicated sustained reductions. The KMRUD policy overall achieved the goal of limiting policy-related drug use and controlling cost increases. And it is recommended that the health department quantify the usage indicator of adjuvant drugs, uniform standards, and apply prescription reviews and dynamic supervision, and other measures to strengthen supervision.

## Introduction

According to a new report, global pharmaceutical spending will exceed $1.5 trillion by 2023 and will grow at a compound annual growth rate of 3%–6% over the next few years (Science, 2019). After achieving universal health coverage in China, pharmaceutical expenditures increased rapidly (Yuan et al., 2021a). Pharmaceuticals expenditures accounted for 30%–40% of total healthcare expenditures in China from 2009 to 2018, much higher than many developed countries, of which the average was less than 15%, such as Sweden (6.6%), Australia (11.9%), the United States (12.4%) in 2018 (OECD, 2021). The high pharmaceutical expenditure is mainly caused by irrational drug use and high drug prices (Ma et al., 2020). In recent years, as local governments across China gradually removed that previously allowed 15% profit margin on drugs, thus the growth rate of medical expenses has been controlled to a certain extent (Fan et al., 2019). However, in China, due to the problems of institutional defects, such as serious information asymmetry and little influence of the principal on the agent, there is no balanced restriction among medical insurance institutions, doctors and medical institutions (Zong et al., 2016). It has led to policy failure to fundamentally change the profit-seeking mechanism of medicine supplements. Some physicians still use drugs unreasonably for profit, including overprescribing adjuvant drugs (Yang et al., 2015).

A World Health Organization (WHO) report noted that, in developing countries and transitional countries, less than 40% of primary care patients in public facilities and 30% in the private sector are treated under standard treatment guidelines (Holloway and Van Dijk, 2011). Unreasonable drug use is a major challenge affecting healthcare systems worldwide. The main reasons for the irrational use of medicines include over-prescribing, over-use of injections, and non-compliance with clinical guidelines (WHO, 2021b). The irrational use of drugs not only increases the medical costs of residents but also leads to adverse drug reactions and increases the incidence and mortality of drug-related diseases (Edwards and Aronson, 2000).

At present, there is no authoritative and clear definition of the concept of adjuvant drugs at the national level in China. In the Medical Subject Headings of the U.S. National Library of Medicine, adjuvant drugs are interpreted as drugs that help to increase the effect of the major therapeutic drugs, or enhance the efficacy of the major therapeutic drug by affecting its absorption, mechanism of action, and metabolism, or contribute to the prevention and treatment of diseases or dysfunction. Adjuvant drugs are often used to prevent or treat major diseases such as tumors, liver diseases, and cardiovascular diseases. In recent years, the use of adjuvant drugs in China has grown rapidly because of the wide application and commercial promotion of adjuvant drugs (Yang et al., 2021). A previous survey showed that 98% of medical institutions in China had unreasonable or irregular use of adjuvant drugs (Han et al., 2016). The key monitoring drugs are an important subset of adjuvant drugs, usually those with high prices, large consumption but unclear therapeutic effects (Li et al., 2022a). The establishment of key monitoring drugs is to reduce the irrational clinical use. Medical institutions monitor the use of these drugs by taking various measures such as prescription review and administrative intervention, so as to standardize the clinical application of adjuvant drugs and control the increase of drug costs.

In 2015, the State Council of the People’s Republic of China issued the Guidance of State Council on Improving the Centralized Procurement of Drugs in Public Hospitals (General Office of the State Council, 2015), which pointed out to focus on tracking and monitoring adjuvant drugs. Since then, to control the unreasonable growth of pharmaceutical expenditures and promote the rational use of adjuvant drugs, local governments have taken a series of policy measures, but they have not worked as expected. There are two major reasons. On the one hand, due to the lack of authoritative and accurate definition of adjuvant drugs, the classification is not clear, for example, a drug has different roles and meanings under different conditions of use, which makes it difficult for medical institutions to determine the objects that need to be monitored (Zhu et al., 2018). On the other hand, due to the lack of systematic regulatory policies specifically for adjuvant drugs, such as unclear drug types and unclear regulatory measures, the control effect is not significant to a certain extent (Wang et al., 2021). The first batch of the catalog of Key Monitoring Drugs (Chemical Drugs and Biological Products) (KMRUD) in China was released by the Medical Administration Bureau of the National Health Commission of the People’s Republic of China in July 2019 (General Office of the National Health Commission, 2019). The document emphasized that monitoring the rational use of drugs is a clear requirement for intensifying the reform of the medical and healthcare system and controlling the unreasonable growth of medical expenses in public hospitals. This is the first time that a national agency has identified drugs requiring key monitoring. A total of 20 adjuvant drugs were listed. The document also proposed to strengthen the clinical application management and the usage monitoring of drugs in the catalog, which pointed out the direction for the supervision of adjuvant drugs. In October 2019, the Health Commission of Hubei Province issued the first batch of the catalog of Key Monitoring and Rational Use Drugs (KMRUD) in Hubei Province (Health Commission of Hubei Province, 2019), which announced 25 adjuvant drugs based on the first batch of the catalog of KMRUD in China. It aims to standardize the clinical application management of key monitoring drugs in medical institutions, so as to control the unreasonable growth of drugs in the catalog. To further intensify the clinical application management of drugs in the catalog, the document requires the public medical institutions to carry out prescription reviews for all drugs in the catalog. The results of prescription review (including the usage and utilization rate) are ranked and publicized internally, and are closely related to the performance pay of doctors and medical staff. In addition, the usage of drugs in the catalog will be included as evaluation indexes in the evaluation and performance appraisal of medical institutions.

At present, only one paper evaluated the impact of the first batch of the catalog of KMRUD in China on the use and expenditure of policy-related drugs (Li et al., 2022a). The research sample is limited to a single hospital, and the object only includes 10 drugs in the KMRUD catalog, which leads to insufficient representativeness of the sample. At present, there are few studies on the evaluation of the implementation effect of the key monitoring drug policy before 2019. The research samples are mostly limited to a single hospital, and the analysis methods are mostly descriptive analysis or simple before-and-after comparison (Li et al., 2018; Zhang et al., 2019; Huang, 2021; Chen, 2022). This study evaluated quantitatively the impact of the first batch of the catalog of KMRUD in Hubei Province on policy-related drug use and expenditures to improve the follow-up batches of the catalog of KMRUD. This study was designed as an Intermittent Time Series (ITS) analysis of the DDDs and spending the policy-related drugs in public medical institutions in Hubei Province, which involved 21 months before the intervention (January 2018-September 2019), and 19 months after the intervention (December 2019-June 2021).

This study was designed to evaluate the impact of the first batch of the catalog of KMRUD during the years 2018–2022 in Hubei Province. The purpose is to provide a basis for the successful implementation of subsequent catalogs of KMRUD, which may promote the standardization of clinical application of related drugs and effectively reduce drug expenses of the patients.

## Methods

### Study setting

This study was undertaken in Hubei province located in central China, with a population of 57.75 million in 2020. The total gross domestic product of the province was 4,344.346 billion (CNY) in 2020, ranking in the middle range of all provinces (Hubei Provincial Statistics Bureau, 2021a). Hubei province consists of 38 counties, with an area of 185.9 thousand square kilometers (Hubei Provincial Statistics Bureau, 2021b). There were 35,447 medical and healthcare institutions in Hubei province, including 1,048 hospitals, and 33,853 primary medical, health institutions and 546 other medical institutions such as public health institutions, maternal and child health hospitals, etc. (Hubei Provincial Statistics Bureau, 2021a). The number of persons engaged in health care institutions in Hubei province is 521.8 thousand, which includes 410.8 thousand medical technical persons (Hubei Provincial Statistics Bureau, 2021b).

### The policy intervention

The first batch of the catalog of KMRUD in Hubei Province was released by the Health Commission of Hubei Province in October 2019 (Health Commission of Hubei Province, 2019). The policy intervention adopted measures to achieve policy goals. Firstly, the catalog of KMRUD was published to the public. Secondly, after the implementation of policy intervention, all public medical institutions established regimens for the management and supervision of KMRUD. The pharmacy department was responsible for training doctors, and for participation in consultations and monitoring (such as prescription review, data collection, and report and feedback). Clinical pharmacists gave their professional advice directly to doctors. The information staff was responsible for the technical implementation of the monitoring system. Additionally, the director of the clinical department was identified as the person responsible for the KMRUD (Health Commission of Hubei Province, 2019). The multidimensional intervention measures were formulated in November 2019 in public medical institutions.

### Data sources

In this study, data on the consumption and spending of policy-related drugs monthly procurement records from January 2018 to June 2021 were obtained from the Drug Centralized Procurement Platform of the Public Resources Trading Center in Hubei Province. All public medical institutions in Hubei Province purchase all drugs through the Drug Centralized Procurement Platform. Each drug procurement record included information on the following variables: medication identifier, generic names, package specifications, pharmaceutical manufacturers, units, price per unit, procurement volumes, procurement expenditures, etc. Since the consumption and spending of each drug were counted monthly separately.

### Identification and classification

The policy-related drugs included the first batch of the catalog of KMRUD in Hubei Province, which consists of a total of 25 drugs (Table 1). The first batch of the catalog of KMRUD was implemented completely in November 2019. In this study, 67450 aggregated procurement records were analyzed, involving 24 drug generic names, and 93 pharmaceutical manufacturers. One drug Ribonucleic Acid (RNA) was excluded from this study because it was not used in Hubei province. In other words, there were no procurement records of this drug’s generic name.

**TABLE 1:** The information of medications on Key Monitoring and Rational Use Drugs (KMRUD) in Hubei Province.

Generic name	Number of products (n)	Number of pharmaceutical manufacturers (n)	Number of hospitals (n)	Number of procurement records (n)
Ganglioside	9	5	301	6979
Cerebroside carnosine	4	2	143	2946
Oxiracetam	7	6	221	4977
Creatine Phosphate Sodium	9	6	102	1819
Calf serum deproteinized	3	1	97	2231
Alprostadil	11	8	230	6002
Troxerutin Cerebroprotein Hydrolyzate	3	1	119	2414
Complex Coenzyme	2	1	51	1235
Salvia Ligustrazine	2	2	230	3929
Invert Sugar Electrolyte	5	2	118	3710
Mouse Nerve Growth Factor	4	4	153	2557
Thymopentin	11	10	60	657
RNA II	3	1	51	941
Edaravone	11	9	207	4711
Osteopeptide	8	7	188	3381
Cerebral Protein Hydrolyzate	7	6	183	1811
Vinpocetine	19	16	233	4428
Calf Blood Deproteinized Extract	9	5	118	1934
Cinepazide Maleate	7	5	105	1473
Guhong Injection	2	1	106	2276
Shenxiong Injection	3	2	178	3269
Lugua Polypeptide Injection	2	1	67	1406
Placenta Polypeptide Injection	1	1	50	1056
Safflower Yellow Injection	3	2	77	1308
Policy-related drugs	145	93	377	67450

### Outcome measures

The outcomes were consumption, spending, and daily costs of policy-related drugs in this study. Drug consumption was defined as Defined Daily Dose (DDD), which is a standard measurement developed by WHO. DDD is defined as the average maintenance daily dose when the drug is used for its major indication in adults. The DDD of the policy-related drugs was identified according to the instructions provided by manufacturers. The drug consumption was calculated by the formula as follows (WHO, 2021a):

$${DDD}_{S}=\overset{n}{\underset{i=1}{\sum }}\left(\frac{package\;specufication\times strengths}{DDD}\times N\right)$$

The spending on policy-related drugs was calculated to reflect the cost in the Chinese national yuan (CNY). The daily costs of drugs were calculated by dividing spending by consumption, also called Defined Daily Dose cost (DDDc) (Guan et al., 2018).

### Statistical analysis

First of all, descriptive analysis was used to present the consumption, spending, and daily costs of policy-related drugs. Graphical displays of the monthly consumption, spending, and DDDc of the policy-related drug were created to observe and describe changes over time from January 2018 to June 2021. The first batch of the catalog of KMRUD in Hubei Province was released in October 2019 and implemented in November 2019. Considering the time point, we compared the corresponding period before (January to September 2018, January to September 2019) and after (January to September 2020) the policy intervention in the descriptive analysis.

An interrupted time-series (ITS) analysis (Linden, 2015) was used to evaluate the impact of the first batch of the catalog of KMRUD policy intervention (Bernal et al., 2017; Zhao et al., 2021). It is a practical quasi-experimental design in the evaluation of population-based health interventions (Craig et al., 2017) and the strongest, quasi-experimental approach for evaluating the longitudinal effects of interventions (Wagner et al., 2002). Because the first batch of the catalog of KMRUD was implemented completely in November 2019, 21 months before the policy intervention (from January 2018 to September 2019), 2 months during the policy intervention (from October 2019 to November 2019), and 19 months after the policy intervention (from December 2019 to 2 June0221) (Health Commission of Hubei Province, 2019). A segmented regression approach was used to evaluate the impact of the policy intervention while controlling covariates (Xiao et al., 2021). The model to be analyzed is as follows:

$$Y_{it}=\beta_{0}+\beta_{1}*\;T_{t}+\beta_{2}*\;X_{t}+\beta_{3}*\;T_{t}\;X_{t}+\beta_{4}*Covid-19+\beta_{5}*SFH+\varepsilon_{it}$$

*Y*_*it*_ is the dependent variable measured at each equally spaced time point *t* (consumption, spending, and daily costs of the policy-related drugs with monthly values). *T*_*t*_ is the number of months since the start of the study. The start of this study is January 2018. *X*_*t*_ is a dummy (indicator) variable representing the implementation of the policy intervention. In this study, *X*_*t*_ is defined as 0 for the period before the implementation of the first batch of the catalog of KMRUD, and 1 for the period after the policy intervention. *T*_*t*_ *X*_*t*_ represents an interaction term between time and intervention (Kontopantelis et al., 2015; Bernal et al., 2018). I represents a drug with the same generic name. This study included 24 generic names of drugs. *COVID-19* was a covariate variable in this study. The government of Hubei Province implements comprehensive and strict control over personnel outflow from January 2020 to April 2020. On April 8, when Wuhan was unsealed, the population flow increased explosively, and the outflow population reached 75.5% of the normal period. Then it entered a stable recovery period of population flow, and the population flow gradually increased (Luo et al., 2020). *COVID-19* is defined as 1 for the period from January 2020 to April 2020. *SFH* was a covariate variable in this study, which represented the Spring Festival Holiday. February 2018 February 2019, January 2020, and February 2021 were the Spring Festival Holiday, which is defined as 1.

The regression coefficient *β*_*0*_ reflects the baseline level of the outcome variable. The regression coefficient *β*_1_ reflects the trajectory trend (slope) before the intervention. The regression coefficient *β*_2_ reflects immediate change for the dependent variable from the pre-intervention to the post-intervention (Linden, 2015; Zhao et al., 2021). The regression coefficient *β*_3_ reflects the change in trend in the outcome variable from the pre-intervention to the post-intervention. In this study, the Spring Festival effect was considered by *β*_5,_ which is the adjustment for Spring Festival Holiday effect. *ε*_*it*_ is an estimate of the random error at observation *Time*_*t*_ (Linden, 2015; Pell et al., 2021).

Additional important issues were also addressed, such as testing for autocorrelation, and robustness testing for interruptions before the true intervention start period. The Durbin-Watson statistic was performed to test for a serial autocorrelation of error terms in the regression models (Bernal et al., 2013). All models were investigated for autocorrelation using the Cumby-Huizinga test autocorrelation (Cumby and Huizinga, 1992). Data management and statistical analysis were performed using Stata 16.0 (Stata Corporation, College Station, TX, United States). Significance was defined as a *p-*value of less than 0.05. The ITS analysis model was used to evaluate the impact of the first batch of the catalog of KMRUD with 95% *CIs* defined based on the linear model predictions.

## Results

### Descriptive analysis of the change in consumption and spending

The information on policy-related drugs in Hubei Province was shown in Table 1.

Graphical displays of the monthly consumption, spending, and DDDc of the policy-related drug were created to observe and describe changes over time from January 2018 to June 2021 (Supplementary Figures S1, S3). After the implementation of the first batch of the catalog of KMRUD, the consumption of policy-related drugs decreased by 83.29% in 2020 (Table 2). The spending on policy-related drugs decreased by 83.93% in 2020 (Table 3). As shown in Table 2 and Table 3, the consumption and spending of Thymopentin decreased by 50.3% and 48.08% after the policy intervention, which both were the minimum value among 24 policy-related drugs. The consumption and spending of Salvia Ligustrazine decreased by 94.96% and 94.96% after the policy intervention, which both were the maximum value among 24 policy-related drugs (Table 2, Table 3).

**TABLE 2:** Descriptive analysis of DDDs on Key Monitoring and Rational Use Drugs (KMRUD) in Hubei Province.

Generic name	Jan. Sep.2018	DDDs (thousand)Jan.-Sep.2019	Jan.-Sep.2020	Growth Rate(%)	
				2019	2020
Ganglioside	2496.76	1401.29	508.66	−43.88	−63.70
Cerebroside carnosine	351.47	252.39	27.81	−28.19	−88.98
Oxiracetam	1158.02	993.87	135.21	−14.18	−86.40
Creatine Phosphate Sodium	205.55	216.30	82.15	5.23	−62.02
Calf serum deproteinized	721.82	368.03	54.03	−49.01	−85.32
Alprostadil	2212.91	2325.09	372.04	5.07	−84.00
Troxerutin Cerebroprotein Hydrolyzate	327.73	346.42	33.28	5.70	−90.39
Complex Coenzyme	425.21	348.66	51.09	−18.00	−85.35
Salvia Ligustrazine	2475.87	1845.84	93.08	−25.45	−94.96
Invert Sugar Electrolyte	776.33	510.21	110.68	−34.28	−78.31
Mouse Nerve Growth Factor	157.86	156.22	44.81	−1.04	−71.32
Thymopentin	890.41	629.61	312.93	−29.29	−50.30
RNA II	239.73	121.92	8.23	−49.15	−93.25
Edaravone	356.91	440.17	109.61	23.33	−75.10
Osteopeptide	537.00	469.05	60.83	−12.65	-87.03
Cerebral Protein Hydrolyzate	449.06	216.66	20.60	−51.75	−90.49
Vinpocetine	1084.88	1241.39	111.46	14.43	−91.02
Calf Blood Deproteinized Extract	353.17	347.40	27.82	−1.64	−91.99
Cinepazide Maleate	210.36	258.51	37.72	22.89	−85.41
Guhong Injection	448.01	507.57	49.94	13.29	−90.16
Shenxiong Injection	944.58	415.25	37.19	−56.04	−91.04
Lugua Polypeptide Injection	434.05	517.75	78.51	19.28	−84.84
Placenta Polypeptide Injection	457.83	266.60	36.98	−41.77	−86.13
Safflower Yellow Injection	304.89	479.88	47.05	57.40	−90.20
**Policy-related drugs**	18020.43	14676.06	2451.71	−18.56	−83.29

**TABLE 3:** Descriptive analysis of spending on Key Monitoring and Rational Use Drugs (KMRUD) in Hubei Province.

Generic name	Jan.-Sep.2018	Spending (million CNY)Jan.-Sep.2019	Jan.-Sep.2020	Growth rate (%)	
				2019	2020
Ganglioside	260.69	123.03	46.22	−52.81	−62.43
Cerebroside carnosine	140.63	101.11	11.17	−28.11	−88.95
Oxiracetam	270.99	233.62	32.64	−13.79	−86.03
Creatine Phosphate Sodium	30.78	29.71	11.80	−3.46	−60.28
Calf serum deproteinized	109.34	56.92	8.15	−47.95	−85.68
Alprostadil	147.17	153.37	26.28	4.21	−82.86
Troxerutin Cerebroprotein Hydrolyzate	74.19	76.86	7.40	3.60	−90.37
Complex Coenzyme	44.73	36.29	5.02	−18.87	−86.18
Salvia Ligustrazine	125.70	93.79	4.73	−25.38	−94.96
Invert Sugar Electrolyte	77.75	52.10	11.43	−32.99	−78.07
Mouse Nerve Growth Factor	35.43	36.17	10.56	2.08	−70,81
Thymopentin	16.32	12.53	6.50	−23.24	−48.08
RNA II	67.41	34.52	2.30	−48.80	−93.34
Edaravone	62.86	73.85	19.43	17.48	−73.70
Osteopeptide	55.58	47.97	5.55	−13.68	−88.43
Cerebral Protein Hydrolyzate	21.82	10.22	1.01	−53.18	−90.11
Vinpocetine	97.59	112.36	10.80	15.13	−90.39
Calf Blood Deproteinized Extract	33.10	32.43	2.72	−2.04	−91,62
Cinepazide Maleate	29.27	35.75	4.82	22.13	−86.53
Guhong Injection	75.29	85.49	8.38	13.55	−90.20
Shenxiong Injection	67.16	32.07	3.10	−52.25	−90.34
Lugua Polypeptide Injection	65.48	78.33	12.37	19.62	−84.20
Placenta Polypeptide Injection	46.96	27.34	3.79	−41.77	−86.13
Safflower Yellow Injection	31.65	48.13	4.78	52.04	−90.06
**Policy-related drugs**	1987.91	1623.94	260.95	−18.31	−83.93

### Impacts on the consumption and spending of policy-related drugs

As shown in Table 4, the consumption of policy-related drugs decreased substantially at the time of the policy intervention implementation. The monthly consumption of policy-related drugs had decreased by 717.80 thousand DDD (*p* < 0.001). The monthly consumption of policy-related drugs had decreased by 249.16 thousand DDDs during COVID-19 (*p =* 0.031). The baseline trend of the monthly consumption before the policy intervention had a significant reduction (*p* < 0.001). The introduction of the first batch of the catalog of KMRUD was associated with a significant decrease in the spending on policy-related drugs in the level (*β*_2_ = -75162.92, *p =* 0.001) (Table 5). The monthly spending on policy-related drugs had decreased by 31155.12 thousand CNY during COVID-19 (*p =* 0.031). The baseline trend of the monthly spending in pre-intervention had a significant reduction (*p* < 0.001) Table 6.

**TABLE 4:** The result of ITS analysis of total consumption on Key Monitoring and Rational Use Drugs in Hubei Province.

Total DDDs (thousand)	Coef	Newey-West Std. Err	t	*p*-value	[95% Conf. Interval]
Baseline trend *β*_*1*_	−32.26	8.37	−3.85	<0.001	(−49.24, −15.27)
Change in level *β*_*2*_	−717.80	185.50	−3.87	<0.001	(−1094.02, −341.59)
Change in trend *β*_*3*_	−10.40	10.86	−0.96	0.345	(−32.43, 11.63)
COVID-19 *β*_*4*_	−249.16	110.81	−2.25	0.031	(−473.90, −24.41)
Spring Festival Effects*β*_*5*_	−134.37	172.13	−0.78	0.440	(−483.47, 214.74)
Constant *β*_*0*_	2155.16	71.52	30.13	<0.001	(2010.11, 2300.22)

**TABLE 5:** The result of ITS analysis of total spending on Key Monitoring and Rational Use Drugs in Hubei Province.

Total Spending (thousand CNY)	Coef	Newey-West Std. Err	t	*p*-value	[95% Conf. Interval]
Baseline trend *β*_*1*_	−3662.19	898.24	−4.08	<0.001	(−5483.90, −1840.48)
Change in level *β*_*2*_	−75162.92	19684.16	−3.82	0.001	(−115084.30, −35241.59)
Change in trend *β*_*3*_	−1389.32	1193.03	−1.16	0.252	(−3808.90, 1030.26)
COVID-19 *β*_*4*_	−31155.12	11959.23	−2.61	0.013	(−55409.57, −6900.67)
Spring Festival Effects*β*_*5*_	−11776.89	16964.86	−0.69	0.492	(−46183.22, 22629.45)
Constant *β*_*0*_	237995.60	8091.93	29.41	<0.001	(221584.40, 254406.80)

**TABLE 6:** The result of ITS analysis of daily cost on Key Monitoring and Rational Use Drugs in Hubei Province.

DDDc (CNY)	Coef	Newey-West Std. Err	t	*p*-value	[95% Conf. Interval]
Baseline trend *β*_*1*_	−0.07	0.14	−0.46	0.645	(−0.35, 0.22)
Change in level *β*_*2*_	16.20	8.60	1.88	0.068	(−1.25, 33.64)
Change in trend *β*_*3*_	−3.57	0.55	−6.43	<0.001	(−4.69, −2.44)
COVID-19 *β*_*4*_	−10.08	7.43	−1.36	0.183	(−25.15, 4.98)
Spring Festival Effects*β*_*5*_	−0.73	4.94	−0.15	0.884	(−10.75, 9.29)
Constant *β*_*0*_	110.82	2.10	52.84	<0.001	(106.57, 115.08)

In each individual ITS analysis of the twenty-four policy-related drugs in this study, ten had a significant reduction in the baseline trend of monthly consumption in pre-intervention (*p* < 0.05). The monthly consumption of seventeen policy-related drugs decreased immediately after the policy intervention implementation (*p* < 0.05), while six policy-related drugs decreased without statistically significant (*p* > 0.05). Ten policy-related drugs had a significant reduction in the trend of monthly consumption in post-implementation months (*p* < 0.05). Five policy-related drugs (including Ganglioside, Calf serum deproteinized, RNA II, Cerebral protein hydrolysate, and Shenxiong injection) had a significant increase trend in monthly DDDs in the post-intervention (*p* < 0.05). Nine policy-related drugs had no significant change in the trend of monthly consumption in post-intervention (*p* > 0.05) (Supplementary Table S1).

Similarly, there was a significant decrease in monthly spending on ten policy-related drugs in the baseline trend before the policy intervention implementation (*p* < 0.05). A sudden significant decrease in monthly spending on eighteen policy-related drugs following the implementation of policy intervention (*p* < 0.05). Ten policy-related drugs had a significant reduction in the trend of monthly spending in post-implementation months (*p* < 0.05). Five policy-related drugs (including Ganglioside, Calf serum deproteinized, RNA II, Cerebral protein hydrolysate, and Shenxiong injection) had a significant increase trend in monthly spending in the post-intervention (*p* < 0.05) (Supplementary Table S1).

### Impacts on the DDDc of policy-related drugs

In the aggregated ITS analysis, the DDDc of policy-related drugs decreased significantly in trend after the implementation of the first batch of the catalog of KMRUD. The downward trend continued (change in the trend of -3.57 CNY per month; *p* < 0.001).

Supplementary Table S2 showed individual ITS analyses for DDDc of all twenty-four policy-related drugs in this study. Only Placenta polypeptide injection had no change in the DDDc. Ganglioside, Edaravone, and Cerebral protein hydrolyzate all had a significant increase in the DDDc following the implementation of policy intervention (*p* < 0.05). Ten policy-related drugs showed a significant decrease trend in the DDDc (*p* < 0.05).

## Discussion

To further strengthen the management of pharmaceutical affairs in medical institutions and promote clinical rational drug use, based on the first batch of the catalog of KMRUD in China, the Health Commission of Hubei Province issued the first Batch of the catalog of KMRUD in Hubei Province, which required all medical institutions to strengthen the supervision of the clinical application of key monitoring drugs. This study preliminarily explores the implementation effect of the KMRUD catalog on policy-related drug use and expenditures in Hubei province. After the implementation of the KMRUD catalog policy, the DDDs and spending on policy-related drugs decreased significantly, by 83.29% and 83.93%, respectively. These results are consistent with the previous results of single-hospital studies focusing on key monitoring drugs policy (Chen et al., 2020; Shen et al., 2020; Gu et al., 2021). These studies indicate that the key monitoring drugs policy has generally achieved policies targets of limiting policy-related drug use and controlling cost increases. A study evaluating the effectiveness of the implementation of the key monitoring drug policy in secondary and tertiary medical institutions in Guangdong Province showed that after the implementation of the policy, the procurement expenditure dropped from 966.4398 million CNY in the first half of 2018 to 653.4824 million CNY in the second half of 2019 (Ke et al., 2021), a decrease of 32.4%. A study on public medical institutions in Huzhou City showed that after the implementation of the key monitoring drug policy, the procurement volume of policy-related drugs decreased by 41.34%, and the procurement expenditure decreased from 99.4186 million CNY to 43.4327 million CNY (Yuan et al., 2021b). These studies have shown that the implementation of the key monitoring drug policy has a positive effect on controlling the excessive and unnecessary use of policy-related drugs.

Before the implementation of the KMRUD catalog policy, DDDs (β_1_ = -32.26 *p* < 0.001) and spending (β_1_ = -3662.19 *p* < 0.001) on policy-related drugs showed a downward trend, while HaiyanLi’s (Li et al., 2022b) study showed that the DDDs (*β*_1_ = 155.78 *p* < 0.05) and spending (*β*_1_ = 1855.34 *p* < 0.05) of the policy-related drug in the baseline part both showed an upward trend. This difference may be explained in part because the Administrative Measures for Adjuvant Drugs in Medical Institutions in Hubei Province (Trial) issued in 2018 has had an expected effect on the use of adjuvant drugs in Hubei province, resulting in a downward trend in the DDDs and spending of policy-related drugs. The research results of Wang Xuemei (Wang et al., 2021) and others showed that after trialing implementation of the policy on adjuvant drug use in Hubei medical institutions in 2018, the total procurement expenditure of policy-related drugs changed from a significant upward trend before the intervention (*β*_1_ = 33.61 *p =* 0.004) to a significant downward trend after the intervention (β_3_ = -83.22 *p* < 0.001). The period after the intervention was consistent with the baseline period of this study (January 2018-October 2019). The ten drugs included in the study are all adjuvant drugs in the KMRUD catalog, thus it can explain the downward trend of the baseline part in this study to a certain extent.

At the policy implementation point, the overall DDDs (β_2_ = -717.80 *p* < 0.001) and spending (β_2_ = -75162.92 *p* < 0.001) of policy-related drugs decreased significantly, and the difference was statistically significant. This result indicated that the KMRUD catalog policy has produced the expected effect on the use of drugs in the catalog in the month of implementation, which also shows that policy regulatory measures have a significant impact on the consumption of policy-related drugs in the short term. However, it is inconsistent with the research results of Haiyan (Li et al., 2022a). Their research showed that in the month of the implementation of the KMRUD catalog policy, the DDDs (β_2_ = -7674.73) and spending (β_2_ = -98363.65) on policy-related drugs decreased but the results were not statistically significant. The possible reasons for this difference are as follows: this study is based on the analysis of the procurement volume and procurement expenditure of all public medical institutions in Hubei province, which is much more than the single hospital drug use in Haiyan Li’s study. In terms of research objects, this study analyzed all 20 drugs in the KMRUD catalog, but only 10 drugs were included in Haiyan Li’s study. Therefore, based on the advantages of a large sample size and a complete variety of drugs, this study can more accurately assess the overall implementation effect of the policy, and the results are more representative.

After the implementation of the KMRUD catalog policy, the long-term trend of the overall DDDs (β_3_ = -10.40 *p =* 0.345) and spending (β_3_ = -1389.32 *p =* 0.252) of policy-related drugs showed a further downward trend, but the results were not statistically significant. This result is consistent with the findings of Haiyan Li et al. (Li et al., 2022a), whose study showed that the DDDs (β_3_ = -430.73) and spending (β_3_ = -4681.93) of policy-related drugs after the implementation of the KMRUD catalog policy were both remarkably decreased but no statistical significance. However, the research of Zhang Wen (Zhang et al., 2021) and others showed that after the implementation of the key monitoring drug policy, the volume (β_3_ = -3.79 *p* < 0.01) and expenditure (β_3_ = -4.256 *p* < 0.01) of policy-related drugs showed a significant downward trend. The possible reason for this difference is that the long-term monitoring of regulatory policies in Hubei Province is not effective enough. The KMRUD policy in Hubei Province did not mention the assessment indicators, evaluation criteria, and other related content of key monitoring drugs. The medical institutions lack a unified supervision method, with differences in the pharmaceutical management level of different medical institutions in the province (Liu et al., 2022), resulting in different levels of understanding of the policy and different implementation efforts during the implementation process. Some hospitals may over-correct to achieve the purpose of cost control, while in some primary medical institutions, overdose is still serious after taking regulatory measures. The ambiguity of this standard affects the regulatory policy efficiency to a certain extent, thus it does not effectively play the role of reducing cost and controlling drug abuse. It is recommended that the health department clarify the assessment indicators and standards, and put forward quantitative indicators for policy-related drugs. The hospital can monitor the proportion and dosage of adjuvant drugs in each department through the information system, then alert doctors when they prescribe drugs that exceed limits. The overall management and control of adjuvant drugs can be realized through the reward and punishment system (Li et al., 2019; Yang et al., 2020). In addition, an intervention study based on PDCA cycle management method proposed to design an exclusive list of key monitoring drugs, which can block the drugs combinations that can not pass the prescription review in advance (Yang et al., 2018). A study on the effect of policy intervention in medical institutions in Yunnan Province of China proposed that medical institutions should implement an early warning notification system for the unusual use of drugs, which can provide early warning for prescriptions with an inappropriate rate of more than 10% in clinical use, so as to promote the rational use of key monitoring drugs (He et al., 2017). But at the same time, it should be noted that the KMRUD catalog policy is not only to reduce the use of a certain drug or a certain type of drug but to make medical institutions and physicians pay more attention to the rationality of the use of adjuvant drugs when prescribing drugs (Zhang et al., 2017). Although these drugs are included in the scope of key monitoring, they still have certain clinical significances that mainly includes assisting conventional treatment measures, improving clinical symptoms, and relieving patients’ pain (Han and Zhao, 2016). A study has proposed to construct a multidisciplinary collaborative clinical rational drug use evaluation system. A group of multidisciplinary experts regularly discusses the irrational drug use in the whole hospital to determine the final reward and punishment results (Li et al., 2022b). Another study proposed that the provincial pharmaceutical quality control center should conduct supervision and inspection in different areas, focusing on the rationality of medical records, such as whether the usage, dosage and indications are appropriate (Liu and Yan, 2017). In this way, medical institutions can place more emphasis on the supervision and management of irrational drug use, thereby the status of rational drug use can be enhanced in the management of key monitoring drugs. After the implementation of the KMRUD catalog policy, the DDDc of policy-related drugs showed a significant downward trend (β_3_ = -3.57 *p* < 0.01), indicating that the burdens of policy-related drugs were reduced, and to a certain extent, the medical costs of the patient’s family and society were reduced.

The research data showed that the monthly procurement volume of 10 policy-related drugs have a significant downward trend after the implementation of the KMRUD catalog policy (*p* < 0.05), and 4 policy-related drugs have a significant upward trend (*p* < 0.05), suggesting that the effect of the KMRUD catalog policy on different kinds of drugs is different. The results of Li Haiyan (Li et al., 2022a) showed that the DDDs and expenditure of 6 drugs in 10 drugs after intervention showed a significant downward trend, which was consistent with the results of this study. Therefore, it is suggested that based on the clinical application status of key monitoring drugs, the drugs in the catalog should be adjusted regularly according to the sales amount and other indicators. Wang et al.'s (Wang et al., 2018) research suggests that the adjuvant drugs with relevant indicators meeting the standard of rational drug use should be removed in time, and drugs with high prices and many adverse reactions should be included in the catalog to ensure the continuous improvement of rational drug use in medical institutions. 5 policy drugs (ganglioside, deproteinized bovine serum, RNA II, cerebroprotein hydrolysate, Shenxiong injection) showed a significant increase in monthly expenditure after intervention (*p* < 0.05). This result is inconsistent with the research results of Chen Xiaofei et al. (Chen et al., 2020) on the intervention of a single hospital. The possible reason is that the sample size is different. The results indicated that the above-mentioned adjuvant drugs need to be further monitored, such as strengthening the training of adjuvant drugs for clinicians in the whole hospital by offering lectures on adjuvant drug knowledge and pharmacists going to clinical departments; increasing the intensity of spot checks and audits of the above drug prescriptions and medical orders and rectifying within a time limit for irrational drug use (Tang and Xi, 2016). According to the efficacy, price, indications, and other factors of adjuvant drugs use, different adjuvant drugs are classified and managed, especially the drugs with poor intervention effects are recommended to be included in the restricted use catalog. After the implementation of the KMRUD policy, the DDDc of 10 policy-related drugs showed a significant downward trend, indicating that this policy spillover effect can reduce the price of some drugs, but for drugs with higher prices, it is necessary to further dynamically monitor their price changes, such as ganglioside, edaravone and cerebroprotein hydrolysate.

The present study had some limitations that should be borne in mind when interpreting the results. First, this study only analyzed the procurement volume, procurement expenditure, and DDDc changes of policy-related drugs at the macro level of medical institutions and did not analyze the dosage and rationality of adjuvant drugs in prescriptions from the individual level of patients. Second, this study used procurement data to analyze usage characteristics, data may be predictive or centralized order bias.

In future research, it is also necessary to further carry out the evaluation of the intervention effect of regulatory policies on the rationality of the use of adjuvant drugs, improve the corresponding indicators and further analyze the dosage and rationality of adjuvant drugs in prescriptions from the individual level of patients. Efforts should be made to pay attention to the rationality of drug use. Moreover, the coverage of policy research should be expanded that obtain a wider range or even nationwide procurement data and carry out the long-term effect evaluation of key monitoring policy to provide a reference for a successful implementation of subsequent catalogs of KMRUD.

## Data Availability

The original contributions presented in the study are included in the article/Supplementary Material, further inquiries can be directed to the corresponding authors.

## Abbreviations

CNY, Chinese Yuan; OECD, Organization for Economic Co-operation and Development; WHO, World Health Organization; KMRUD, Key Monitoring and Rational Use Drugs; PRC, People’s Republic of China; NHSA, National Healthcare Security Administration; DDD, Defined Daily Dose; DDDs, Defined Daily Doses; DDDc, Defined Daily Dose cost; ITS, Interrupted Time Series; RNA, Ribonucleic Acid.
