# Supplementary material for: The impact of key monitoring policy on the usage of policy-related drugs in Hubei Province, China
Source: Front Pharmacol. 2023 Feb 15;14:1088723. doi: 10.3389/fphar.2023.1088723 (PMC9976703; doi:10.3389/fphar.2023.1088723)
Supplement: Supplementary file 1 [file Table1.DOCX]

**Supplementary File 1**

**Figure S1. The monthly consumption of on Key Monitoring and Rational Use Drugs (KMRUD）in Hubei province**

**Figure S2. The monthly spending of on Key Monitoring and Rational Use Drugs (KMRUD）in Hubei province**

**Figure S3. The monthly DDDc of on Key Monitoring and Rational Use Drugs (KMRUD）in Hubei province**

**Table S1. The result of the ITS analysis of consumption and spending on Key Monitoring and Rational Use Drugs (KMRUD）in Hubei province**

| Categories | | DDDs (thousand） | | Spending (thousand CNY） | | |
| --- | --- | --- | --- | --- | --- | --- |
|  |  | Coef.( 95%*C.I.*) | *p*-value | Coef.( 95%*C.I.*) | | *p*-value |
| **Ganglioside** | |  |  | |  |  |
|  | Baseline trend *β_1_* | -9755.35(-11595.31,-7915.39) | <0.001 | | -1207.45(-1362.54,-1052.36) | <0.001 |
|  | Change in level *β_2_* | -24674.30(-55135.71,5787.10) | 0.109 | | 23.47(-2709.86,2756.81) | 0.986 |
|  | Change in trend *β_3_* | 6530.82(4544.12,8517.51) | <0.001 | | 814.78(644.56,985.00) | <0.001 |
|  | COVID-19 *β_4_* | -28727.79(-55017.61,-2437.98) | 0.033 | | -2394.11(-5043.68,255.47) | 0.075 |
|  | Spring Festival Effects *β_5_* | -29526.81(-81200.60,22146.99) | 0.254 | | -3461.68(-8652.92,1729.56) | 0.185 |
|  | Constant *β_0_* | 318031.90(299938.40,336125.50) | <0.001 | | 33870.93(32044.19,35697.67) | <0.001 |
| **Cerebroside carnosine** | |  |  | |  |  |
|  | Baseline trend *β_1_* | -956.67(-1410.29,-503.05) | <0.001 | | -388.78(-563.12,-214.43) | <0.001 |
|  | Change in level *β_2_* | -7889.38(-15101.23,-677.52) | 0.033 | | -4205.13(-7456.63,-953.63) | 0.013 |
|  | Change in trend *β_3_* | -330.87(-841.05,179.30) | 0.195 | | 68.66(-145.70,283.02) | 0.520 |
|  | COVID-19 *β_4_* | -6303.07(-9308.48,-3297.66) | <0.001 | | -2171.17(-3689.22,-653.13) | 0.006 |
|  | Spring Festival Effects *β_5_* | 5404.76(1705.05,9104.47) | 0.006 | | 1571.22(92.02,3050.42) | 0.038 |
|  | Constant *β_0_* | 42750.32(37330.15,48170.49) | <0.001 | | 17204.92(15119.71,19290.12) | <0.001 |
| **Oxiracetam** | |  |  | |  |  |
|  | Baseline trend *β_1_* | -1647.71(-2834.49,-460.92) | 0.008 | | -380.28(-671.69,-88.87) | 0.012 |
|  | Change in level *β_2_* | -55658.45(-79879.80,-31437.10) | <0.001 | | -12656.63(-18217.55,-7095.71) | <0.001 |
|  | Change in trend *β_3_* | -871.06(-2472.48,730.35) | 0.277 | | -260.46(-645.33,124.41) | 0.178 |
|  | COVID-19 *β_4_* | -16889.29(-32135.83,-1642.76) | 0.031 | | -4192.72(-7750.60,-634.84) | 0.022 |
|  | Spring Festival Effects *β_5_* | -6941.52(-26249.51,12366.46) | 0.471 | | -1541.91(-6030.93,2947.10) | 0.491 |
|  | Constant *β_0_* | 134642.30(120417.30,148867.30) | <0.001 | | 31483.98(27840.00,35127.97) | <0.001 |
| **Creatine Phosphate Sodium** | |  |  | |  |  |
|  | Baseline trend *β_1_* | 8.64(-439.31,456.59) | 0.969 | | -19.66(-83.22,43.90) | 0.534 |
|  | Change in level *β_2_* | -10461.14(-17439.83,-3482.45) | 0.004 | | -1264.46(-2255.36,-273.57) | 0.014 |
|  | Change in trend *β_3_* | -560.62(-1023.71,-97.52) | 0.019 | | -74.01(-143.39,-4.63) | 0.037 |
|  | COVID-19 *β_4_* | -1448.70(-6222.08,3324.68) | 0.542 | | -229.10(-916.79,458.58) | 0.504 |
|  | Spring Festival Effects *β_5_* | -240.61(-3204.23,2723.02) | 0.870 | | -90.16(-489.98,309.66) | 0.650 |
|  | Constant *β_0_* | 23448.28(20001.87,26894.69) | <0.001 | | 3590.43(3076.27,4104.59) | <0.001 |
| **Calf serum deproteinized** | |  |  | |  |  |
|  | Baseline trend *β_1_* | -3421.75(-5176.07,-1667.43) | <0.001 | | -502.42(-746.13,-258.70) | <0.001 |
|  | Change in level *β_2_* | -1997.01(-16765.24,12771.22) | 0.784 | | -876.25(-2978.94,1226.44) | 0.404 |
|  | Change in trend *β_3_* | 1999.28(344.47,3654.09) | 0.020 | | 344.13(89.09,599.18) | 0.010 |
|  | COVID-19 *β_4_* | -6509.08(-12306.08,-712.09) | 0.029 | | -989.43(-1738.24,-240.63) | 0.011 |
|  | Spring Festival Effects *β_5_* | -4279.62(-24286.67,15727.43) | 0.665 | | -409.63(-2289.86,1470.60) | 0.661 |
|  | Constant *β_0_* | 93386.62(67438.91,119334.30) | <0.001 | | 14052.87(10476.63,17629.11) | <0.001 |
| **Alprostadil** | |  |  | |  |  |
|  | Baseline trend *β_1_* | 647.44(-3467.10,4761.98) | 0.751 | | 28.48(-245.02,301.98) | 0.834 |
|  | Change in level *β_2_* | -147237.80(-222916.30,-71559.34) | <0.001 | | -9279.44(-14143.72,-4415.15) | <0.001 |
|  | Change in trend *β_3_* | -6866.71(-11839.63,-1893.79) | 0.008 | | -476.78(-797.71,-155.84) | 0.005 |
|  | COVID-19 *β_4_* | -32858.87(-73603.96,7886.22) | 0.111 | | -2226.73(-4808.36,354.89) | 0.089 |
|  | Spring Festival Effects *β_5_* | -17988.37(-92091.43,56114.70) | 0.625 | | -1292.73(-6048.66,3463.20) | 0.585 |
|  | Constant *β_0_* | 250330.00(207929.30,292730.70) | <0.001 | | 16724.55(13902.87,19546.24) | <0.001 |
| **Troxerutin Cerebroprotein Hydrolyzate** | | |  | |  |  |
|  | Baseline trend *β_1_* | -75.09(-864.49,714.31) | 0.847 | | -27.76(-197.25,141.72) | 0.742 |
|  | Change in level *β_2_* | -16448.59(-28107.63,-4789.56) | 0.007 | | -4515.51(-7432.14,-1598.89) | 0.003 |
|  | Change in trend *β_3_* | -1757.33(-2637.65,-877.02) | <0.001 | | -205.23(-412.08,1.62) | 0.052 |
|  | COVID-19 *β_4_* | -7211.07(-12145.07,-2277.07) | 0.006 | | -1484.34(-2855.77,-112.90) | 0.035 |
|  | Spring Festival Effects *β_5_* | -463.93(-7240.14,6312.27) | 0.889 | | 79.58(-919.93,1079.10) | 0.873 |
|  | Constant *β_0_* | 38453.19(30217.49,46688.89) | <0.001 | | 8690.08(6947.77,10432.40) | <0.001 |
| **Complex Coenzyme** | |  |  | |  |  |
|  | Baseline trend *β_1_* | -791.09(-1664.40,82.22) | 0.074 | | -87.53(-177.60,2.55) | 0.056 |
|  | Change in level *β_2_* | -18270.97(-33442.38,-3099.57) | 0.020 | | -2075.08(-3611.31,-538.84) | 0.010 |
|  | Change in trend *β_3_* | -524.39(-1409.69,360.91) | 0.235 | | -9.72(-104.31,84.87) | 0.836 |
|  | COVID-19 *β_4_* | -5154.74(-8812.06,-1497.41) | 0.007 | | -530.49(-965.49,-95.48) | 0.018 |
|  | Spring Festival Effects *β_5_* | -4067.35(-10979.29,2844.60) | 0.238 | | -212.82(-733.65,308.00) | 0.413 |
|  | Constant *β_0_* | 52620.06(42345.85,62894.27) | <0.001 | | 5503.63(4465.60,6541.67) | <0.001 |
| **Salvia Ligustrazine** | |  |  | |  |  |
|  | Baseline trend *β_1_* | -5928.58(-7938.39,-3918.78) | <0.001 | | -289.69(-391.35,-188.04) | <0.001 |
|  | Change in level *β_2_* | -66136.72(-114770.50,-17502.97) | 0.009 | | -4851.57(-7837.33,-1865.80) | 0.002 |
|  | Change in trend *β_3_* | -4546.79(-8276.13,-817.45) | 0.019 | | 25.77(-152.26,203.79) | 0.771 |
|  | COVID-19 *β_4_* | -38379.64(-66214.40,-10544.87) | 0.009 | | -1973.19(-3799.90,-146.48) | 0.035 |
|  | Spring Festival Effects *β_5_* | -34454.83(-87220.20,18310.55) | 0.192 | | -842.80(-2971.29,1285.69) | 0.427 |
|  | Constant *β_0_* | 297891.80(279986.50,315797.20) | <0.001 | | 14972.15(14024.22,15920.08) | <0.001 |
| **Invert Sugar Electrolyte** | |  |  | |  |  |
|  | Baseline trend *β_1_* | -2184.49(-3110.23,-1258.76) | <0.001 | | -208.66(-301.87,-115.45) | <0.001 |
|  | Change in level *β_2_* | -14935.12(-28544.58,-1325.65) | 0.032 | | -1496.81(-2867.41,-126.21) | 0.033 |
|  | Change in trend *β_3_* | 476.62(-718.81,1672.05) | 0.424 | | 22.18(-100.05,144.41) | 0.715 |
|  | COVID-19 *β_4_* | -11344.19(-23130.84,442.45) | 0.059 | | -1203.31(-2410.30,3.69) | 0.051 |
|  | Spring Festival Effects *β_5_* | -6318.19(-29908.79,17272.40) | 0.590 | | -600.80(-2992.53,1790.94) | 0.614 |
|  | Constant *β_0_* | 92801.49(80846.78,104756.20) | <0.001 | | 9243.84(8026.74,10460.95) | <0.001 |
| **Mouse Nerve Growth Factor** | |  |  | |  |  |
|  | Baseline trend *β_1_* | -40.54(-247.47,166.40) | 0.693 | | -1.51(-50.02,47.00) | 0.950 |
|  | Change in level *β_2_* | -6273.17(-12589.64,43.31) | 0.052 | | -1352.61(-2662.31,-42.90) | 0.043 |
|  | Change in trend *β_3_* | -314.07(-763.45,135.32) | 0.165 | | -114.63(-208.61,-20.64) | 0.018 |
|  | COVID-19 *β_4_* | -5584.09(-9743.72,-1424.46) | 0.010 | | -1215.43(-2051.89,-378.98) | 0.006 |
|  | Spring Festival Effects *β_5_* | 1404.09(-1686.13,4494.30) | 0.363 | | 116.55(-350.62,583.71) | 0.616 |
|  | Constant *β_0_* | 17836.47(16314.15,19358.79) | <0.001 | | 3999.26(3652.85,4345.67) | <0.001 |
| **Thymopentin** | |  |  | |  |  |
|  | Baseline trend *β_1_* | -1711.51(-3618.70,195.67) | 0.077 | | -24.07(-60.90,12.76) | 0.193 |
|  | Change in level *β_2_* | -34053.45(-66384.25,-1722.64) | 0.040 | | -524.49(-982.90,-66.08) | 0.026 |
|  | Change in trend *β_3_* | 1141.13(-1344.52,3626.78) | 0.355 | | -18.22(-57.83,21.38) | 0.357 |
|  | COVID-19 *β_4_* | 12666.97(-756.29,26090.24) | 0.063 | | 387.74(44.70,730.78) | 0.028 |
|  | Spring Festival Effects *β_5_* | -13659.36(-28410.56,1091.84) | 0.068 | | -440.19(-758.88,-121.50) | 0.008 |
|  | Constant *β_0_* | 107001.50(80517.17,133485.80) | <0.001 | | 1884.61(1380.93,2388.30) | <0.001 |
| **RNA II** | |  |  | |  |  |
|  | Baseline trend *β_1_* | -1207.60(-1918.47,-496.73) | 0.002 | | -337.72(-523.83,-151.62) | 0.001 |
|  | Change in level *β_2_* | -1362.90(-7268.47,4542.68) | 0.640 | | -647.71(-2176.14,880.72) | 0.396 |
|  | Change in trend *β_3_* | 814.32(119.27,1509.37) | 0.023 | | 266.24(71.09,461.38) | 0.009 |
|  | COVID-19 *β_4_* | -2097.47(-4057.78,-137.16) | 0.037 | | -525.62(-1026.52,-24.71) | 0.040 |
|  | Spring Festival Effects *β_5_* | 670.78(-5505.66,6847.22) | 0.826 | | 124.10(-946.63,1194.84) | 0.815 |
|  | Constant *β_0_* | 31500.56(20605.24,42395.88) | <0.001 | | 8858.60(6001.73,11715.47) | <0.001 |
| **Edaravone** | |  |  | |  |  |
|  | Baseline trend *β_1_* | 538.72(-194.74,1272.19) | 0.145 | | 60.37(-67.94,188.69) | 0.346 |
|  | Change in level *β_2_* | -22601.57(-36116.44,-9086.70) | 0.002 | | -3264.53(-5616.67,-912.38) | 0.008 |
|  | Change in trend *β_3_* | -2077.57(-2885.95,-1269.19) | <0.001 | | -348.72(-491.40,-206.05) | <0.001 |
|  | COVID-19 *β_4_* | -8447.92(-17271.78,375.95) | 0.060 | | -1490.62(-3315.02,333.78) | 0.106 |
|  | Spring Festival Effects *β_5_* | -3246.27(-9658.01,3165.46) | 0.311 | | -727.28(-1924.05,469.49) | 0.226 |
|  | Constant *β_0_* | 39467.42(34144.11,44790.72) | <0.001 | | 7075.87(6155.58,7996.16) | <0.001 |
| **Osteopeptide** | |  |  | |  |  |
|  | Baseline trend *β_1_* | -888.00(-1799.54,23.55) | 0.056 | | -92.37(-188.26,3.51) | 0.059 |
|  | Change in level *β_2_* | -25473.23(-40628.29,-10318.17) | 0.002 | | -2925.32(-4439.61,-1411.04) | <0.001 |
|  | Change in trend *β_3_* | -337.91(-1251.08,575.26) | 0.458 | | -15.64(-109.08,77.80) | 0.736 |
|  | COVID-19 *β_4_* | -7634.47(-14162.61,-1106.33) | 0.023 | | -652.43(-1283.64,-21.21) | 0.043 |
|  | Spring Festival Effects *β_5_* | -5979.72(-16251.99,4292.56) | 0.246 | | -736.50(-2061.37,588.36) | 0.267 |
|  | Constant *β_0_* | 65122.38(56163.66,74081.10) | <0.001 | | 6720.19(5791.46,7648.92) | <0.001 |
| **Cerebral Protein Hydrolyzate** | |  |  | |  |  |
|  | Baseline trend *β_1_* | -2249.46(-2729.04,-1769.89) | <0.001 | | -112.29(-135.47,-89.12) | <0.001 |
|  | Change in level *β_2_* | -7527.46(-13340.59,-1714.33) | 0.013 | | -318.00(-596.10,-39.91) | 0.026 |
|  | Change in trend *β_3_* | 1983.19(1491.53,2474.85) | <0.001 | | 99.13(75.33,122.93) | <0.001 |
|  | COVID-19 *β_4_* | 269.61(-2957.13,3496.34) | 0.866 | | 14.32(-144.90,173.54) | 0.856 |
|  | Spring Festival Effects *β_5_* | -4162.84(-12505.82,4180.15) | 0.318 | | -206.40(-622.70,209.89) | 0.321 |
|  | Constant *β_0_* | 59533.18(53454.59,65611.78) | <0.001 | | 2910.64(2610.31,3210.97) | <0.001 |
| **Vinpocetine** | |  |  | |  |  |
|  | Baseline trend *β_1_* | 918.36(-431.16,2267.88) | 0.176 | | 92.66(-29.20,214.53) | 0.132 |
|  | Change in level *β_2_* | -81799.09(-119910.70,-43687.49) | <0.001 | | -7391.28(-10721.87,-4060.69) | <0.001 |
|  | Change in trend *β_3_* | -4751.97(-6920.00,-2583.94) | <0.001 | | -443.03(-630.04,-256.02) | <0.001 |
|  | COVID-19 *β_4_* | -24868.88(-47298.75,-2439.01) | 0.031 | | -2215.86(-4212.93,-218.80) | 0.031 |
|  | Spring Festival Effects *β_5_* | -14010.94(-40788.49,12766.61) | 0.296 | | -1371.80(-3911.69,1168.10) | 0.281 |
|  | Constant *β_0_* | 123348.60(109917.40,136779.70) | <0.001 | | 11006.64(9820.51,12192.77) | <0.001 |
| **Calf Blood Deproteinized Extract** | |  |  | |  |  |
|  | Baseline trend *β_1_* | -157.42(-860.70,545.87) | 0.651 | | -13.23(-76.39,49.93) | 0.673 |
|  | Change in level *β_2_* | -19478.71(-33339.16,-5618.26) | 0.007 | | -2145.15(-3452.03,-838.27) | 0.002 |
|  | Change in trend *β_3_* | -1481.22(-2456.26,-506.18) | 0.004 | | -83.50(-160.50,-6.51) | 0.034 |
|  | COVID-19 *β_4_* | -5940.20(-13254.32,1373.93) | 0.108 | | -548.93(-1246.50,148.63) | 0.119 |
|  | Spring Festival Effects *β_5_* | -10871.62(-25341.81,3598.57) | 0.136 | | -597.41(-1664.22,469.40) | 0.264 |
|  | Constant *β_0_* | 42945.59(34501.60,51389.59) | <0.001 | | 3956.05(3216.60,4695.49) | <0.001 |
| **Cinepazide Maleate** | |  |  | |  |  |
|  | Baseline trend *β_1_* | 507.92(-93.91,1109.75) | 0.095 | | 65.59(-21.72,152.90) | 0.136 |
|  | Change in level *β_2_* | -17552.67(-24888.98,-10216.37) | <0.001 | | -2682.32(-3765.88,-1598.76) | <0.001 |
|  | Change in trend *β_3_* | -1516.99(-2121.84,-912.14) | <0.001 | | -164.97(-256.22,-73.71) | 0.001 |
|  | COVID-19 *β_4_* | -5513.13(-7913.63,-3112.63) | <0.001 | | -709.15(-1060.76,-357.53) | <0.001 |
|  | Spring Festival Effects *β_5_* | -2376.43(-8772.58,4019.73) | 0.453 | | -220.78(-813.20,371.65) | 0.455 |
|  | Constant *β_0_* | 20899.59(13503.47,28295.70) | <0.001 | | 2947.73(1847.34,4048.12) | <0.001 |
| **Guhong Injection** | |  |  | |  |  |
|  | Baseline trend *β_1_* | 447.12(-33.33,927.57) | 0.067 | | 83.31(7.31,159.30) | 0.033 |
|  | Change in level *β_2_* | -16170.94(-29190.16,-3151.72) | 0.017 | | -4464.54(-7530.00,-1399.07) | 0.006 |
|  | Change in trend *β_3_* | -4441.64(-5674.05,-3209.23) | <0.001 | | -440.63(-641.34,-239.92) | <0.001 |
|  | COVID-19 *β_4_* | -15267.96(-25246.39,-5289.54) | 0.004 | | -2452.73(-4595.14,-310.31) | 0.026 |
|  | Spring Festival Effects *β_5_* | -3917.88(-15826.43,7990.67) | 0.506 | | -117.36(-1644.88,1410.17) | 0.877 |
|  | Constant *β_0_* | 48999.81(43838.08,54161.53) | <0.001 | | 8148.34(7395.60,8901.07) | <0.001 |
| **Shenxiong Injection** | |  |  | |  |  |
|  | Baseline trend *β_1_* | -4611.72(-5502.13,-3721.31) | <0.001 | | -299.13(-358.54,-239.73) | <0.001 |
|  | Change in level *β_2_* | 261.06(-11254.07,11776.20) | 0.963 | | -706.36(-1705.04,292.31) | 0.160 |
|  | Change in trend *β_3_* | 2124.73(986.36,3263.11) | 0.001 | | 200.26(115.66,284.87) | <0.001 |
|  | COVID-19 *β_4_* | -3854.23(-14382.06,6673.61) | 0.460 | | -366.61(-1192.15,458.92) | 0.374 |
|  | Spring Festival Effects *β_5_* | -12086.10(-40465.63,16293.43) | 0.390 | | -373.81(-1848.36,1100.75) | 0.610 |
|  | Constant *β_0_* | 121836.10(111638.00,132034.20) | <0.001 | | 8501.22(7797.99,9204.44) | <0.001 |
| **Lugua Polypeptide Injection** | |  |  | |  |  |
|  | Baseline trend *β_1_* | 250.99(-943.99,1445.98) | 0.670 | | 58.23(-116.93,233.39) | 0.504 |
|  | Change in level *β_2_* | -16509.57(-34591.82,1572.67) | 0.072 | | -3608.89(-6912.60,-305.18) | 0.033 |
|  | Change in trend *β_3_* | -3624.46(-5147.87,-2101.05) | <0.001 | | -385.07(-600.15,-170.00) | 0.001 |
|  | COVID-19 *β_4_* | -14919.03(-24318.05,-5520.01) | 0.003 | | -2330.96(-3887.11,-774.80) | 0.004 |
|  | Spring Festival Effects *β_5_* | -8649.04(-31964.87,14666.79) | 0.454 | | -574.37(-2831.80,1683.06) | 0.609 |
|  | Constant *β_0_* | 51833.62(39163.72,64503.53) | <0.001 | | 7615.47(5794.84,9436.10) | <0.001 |
| **Placenta Polypeptide Injection** | |  |  | |  |  |
|  | Baseline trend *β_1_* | -1815.57(-2759.36,-871.79) | <0.001 | | -184.97(-276.78,-93.15) | <0.001 |
|  | Change in level *β_2_* | -1131.19(-11968.27,9705.90) | 0.832 | | -444.15(-1621.74,733.44) | 0.449 |
|  | Change in trend *β_3_* | 299.13(-652.63,1250.89) | 0.525 | | 86.45(-14.04,186.95) | 0.090 |
|  | COVID-19 *β_4_* | -6469.53(-9483.81,-3455.25) | <0.001 | | -627.63(-1030.79,-224.47) | 0.003 |
|  | Spring Festival Effects *β_5_* | -1793.03(-9423.40,5837.34) | 0.634 | | -76.49(-593.06,440.08) | 0.766 |
|  | Constant *β_0_* | 57646.71(45701.81,69591.60) | <0.001 | | 5895.15(4740.45,7049.86) | <0.001 |
| **Safflower Yellow Injection** | |  |  | |  |  |
|  | Baseline trend *β_1_* | 1383.96(308.70,2459.22) | 0.014 | | 126.70(28.18,225.23) | 0.013 |
|  | Change in level *β_2_* | -30149.26(-47941.54,-12356.98) | 0.002 | | -3490.17(-5378.27,-1602.07) | 0.001 |
|  | Change in trend *β_3_* | -3942.86(-5268.48,-2617.24) | <0.001 | | -276.31(-395.54,-157.09) | <0.001 |
|  | COVID-19 *β_4_* | -10289.85(-17807.33,-2772.36) | 0.009 | | -1026.61(-1886.54,-166.69) | 0.021 |
|  | Spring Festival Effects *β_5_* | 1852.49(-12056.17,15761.15) | 0.787 | | 226.58(-565.38,1018.55) | 0.565 |
|  | Constant *β_0_* | 29636.84(21084.91,38188.77) | <0.001 | | 3138.41(2385.97,3890.84) | <0.001 |

**Table S2. The result of the ITS analysis of DDDc on Key Monitoring and Rational Use Drugs in Hubei Province**

| Categories | | DDDc (CNY) | |
| --- | --- | --- | --- |
|  |  | Coef.( 95%*C.I.*) | *p*-value |
| **Ganglioside** | |  |  |
|  | Baseline trend *β_1_* | -1.32(-1.71,-0.93) | <0.001 |
|  | Change in level *β_2_* | 22.48(4.83,40.13) | 0.014 |
|  | Change in trend *β_3_* | -2.01(-3.07,-0.95) | <0.001 |
|  | COVID-19 *β_4_* | 5.59(-6.75,17.94) | 0.364 |
|  | Spring Festival Effects *β_5_* | -9.11(-16.54,-1.68) | 0.018 |
|  | Constant *β_0_* | 110.42(106.25,114.59) | <0.001 |
| **Cerebroside carnosine** | |  |  |
|  | Baseline trend *β_1_* | 0.05(0.00,0.10) | 0.069 |
|  | Change in level *β_2_* | -0.30(-1.60,1.00) | 0.643 |
|  | Change in trend *β_3_* | 0.06(-0.08,0.19) | 0.375 |
|  | COVID-19 *β_4_* | 0.26(-1.20,1.72) | 0.719 |
|  | Spring Festival Effects *β_5_* | 0.91(0.27,1.54) | 0.007 |
|  | Constant *β_0_* | 399.97(399.30,400.63) | <0.001 |
| **Oxiracetam** | |  |  |
|  | Baseline trend *β_1_* | -0.08(-0.45,0.30) | 0.678 |
|  | Change in level *β_2_* | 46.47(-5.83,98.77) | 0.080 |
|  | Change in trend *β_3_* | -8.85(-12.14,-5.57) | <0.001 |
|  | COVID-19 *β_4_* | -3.59(-42.88,35.70) | 0.854 |
|  | Spring Festival Effects *β_5_* | -13.26(-29.59,3.07) | 0.108 |
|  | Constant *β_0_* | 235.57(230.62,240.53) | <0.001 |
| **Creatine Phosphate Sodium** | |  |  |
|  | Baseline trend *β_1_* | -1.10(-1.52,-0.68) | <0.001 |
|  | Change in level *β_2_* | 30.03(-10.35,70.41) | 0.140 |
|  | Change in trend *β_3_* | -4.18(-6.68,-1.68) | 0.002 |
|  | COVID-19 *β_4_* | 0.59(-28.99,30.17) | 0.968 |
|  | Spring Festival Effects *β_5_* | -11.43(-24.14,1.28) | 0.076 |
|  | Constant *β_0_* | 155.38(150.40,160.36) | <0.001 |
| **Calf serum deproteinized** | |  |  |
|  | Baseline trend *β_1_* | 0.16(-0.17,0.50) | 0.329 |
|  | Change in level *β_2_* | -3.29(-8.30,1.73) | 0.190 |
|  | Change in trend *β_3_* | -0.04(-0.56,0.49) | 0.885 |
|  | COVID-19 *β_4_* | -3.09(-5.72,-0.45) | 0.024 |
|  | Spring Festival Effects *β_5_* | -1.45(-3.82,0.91) | 0.218 |
|  | Constant *β_0_* | 151.81(148.70,154.92) | <0.001 |
| **Alprostadil** | |  |  |
|  | Baseline trend *β_1_* | -0.10(-0.19,-0.01) | 0.023 |
|  | Change in level *β_2_* | 11.37(-1.64,24.38) | 0.085 |
|  | Change in trend *β_3_* | -1.45(-2.26,-0.64) | 0.001 |
|  | COVID-19 *β_4_* | -0.79(-10.51,8.92) | 0.869 |
|  | Spring Festival Effects *β_5_* | -3.63(-8.08,0.83) | 0.107 |
|  | Constant *β_0_* | 67.43(66.20,68.66) | <0.001 |
| **Troxerutin Cerebroprotein Hydrolyzate** | |  |  |
|  | Baseline trend *β_1_* | -0.42(-0.64,-0.20) | 0.001 |
|  | Change in level *β_2_* | 1.16(-5.70,8.01) | 0.733 |
|  | Change in trend *β_3_* | 0.52(-0.33,1.38) | 0.220 |
|  | COVID-19 *β_4_* | 6.93(0.33,13.53) | 0.040 |
|  | Spring Festival Effects *β_5_* | -6.25(-17.62,5.12) | 0.270 |
|  | Constant *β_0_* | 227.87(225.13,230.61) | <0.001 |
| **Complex Coenzyme** | |  |  |
|  | Baseline trend *β_1_* | -0.08(-0.53,0.38) | 0.733 |
|  | Change in level *β_2_* | 1.50(-4.20,7.19) | 0.594 |
|  | Change in trend *β_3_* | -0.87(-1.47,-0.26) | 0.007 |
|  | COVID-19 *β_4_* | -2.25(-10.64,6.13) | 0.586 |
|  | Spring Festival Effects *β_5_* | 6.17(-3.76,16.10) | 0.214 |
|  | Constant *β_0_* | 104.84(99.21,110.47) | <0.001 |
| **Salvia Ligustrazine** | |  |  |
|  | Baseline trend *β_1_* | 0.00(0.00,0.01) | 0.168 |
|  | Change in level *β_2_* | -0.05(-0.12,0.01) | 0.089 |
|  | Change in trend *β_3_* | 0.01(0.00,0.02) | 0.005 |
|  | COVID-19 *β_4_* | -0.03(-0.14,0.08) | 0.581 |
|  | Spring Festival Effects *β_5_* | -0.04(-0.25,0.17) | 0.707 |
|  | Constant *β_0_* | 50.77(50.71,50.83) | <0.001 |
| **Invert Sugar Electrolyte** | |  |  |
|  | Baseline trend *β_1_* | 0.15(0.06,0.25) | 0.003 |
|  | Change in level *β_2_* | 10.11(-1.65,21.88) | 0.090 |
|  | Change in trend *β_3_* | -2.39(-3.11,-1.67) | <0.001 |
|  | COVID-19 *β_4_* | -1.01(-9.84,7.83) | 0.819 |
|  | Spring Festival Effects *β_5_* | -2.29(-7.24,2.66) | 0.355 |
|  | Constant *β_0_* | 99.66(98.60,100.73) | <0.001 |
| **Mouse Nerve Growth Factor** | |  |  |
|  | Baseline trend *β_1_* | 0.43(0.17,0.69) | 0.002 |
|  | Change in level *β_2_* | 22.70(-15.10,60.50) | 0.231 |
|  | Change in trend *β_3_* | -6.77(-9.09,-4.45) | <0.001 |
|  | COVID-19 *β_4_* | 10.66(-17.46,38.78) | 0.447 |
|  | Spring Festival Effects *β_5_* | -13.64(-26.34,-0.93) | 0.036 |
|  | Constant *β_0_* | 224.46(221.07,227.86) | <0.001 |
| **Thymopentin** | |  |  |
|  | Baseline trend *β_1_* | 0.15(-0.01,0.31) | 0.070 |
|  | Change in level *β_2_* | -1.52(-5.19,2.14) | 0.402 |
|  | Change in trend *β_3_* | 0.11(-0.15,0.37) | 0.380 |
|  | COVID-19 *β_4_* | 1.74(-2.17,5.65) | 0.370 |
|  | Spring Festival Effects *β_5_* | -4.61(-6.99,-2.22) | <0.001 |
|  | Constant *β_0_* | 18.01(16.72,19.30) | <0.001 |
| **RNA II** | |  |  |
|  | Baseline trend *β_1_* | 0.08(-0.20,0.36) | 0.579 |
|  | Change in level *β_2_* | -2.66(-7.21,1.89) | 0.241 |
|  | Change in trend *β_3_* | -0.31(-0.77,0.14) | 0.167 |
|  | COVID-19 *β_4_* | 0.32(-1.98,2.62) | 0.776 |
|  | Spring Festival Effects *β_5_* | -1.39(-4.38,1.60) | 0.349 |
|  | Constant *β_0_* | 281.82(279.34,284.30) | <0.001 |
| **Edaravone** | |  |  |
|  | Baseline trend *β_1_* | -0.85(-1.24,-0.45) | <0.001 |
|  | Change in level *β_2_* | 35.25(7.63,62.88) | 0.014 |
|  | Change in trend *β_3_* | -4.24(-5.95,-2.53) | <0.001 |
|  | COVID-19 *β_4_* | -5.29(-38.13,27.55) | 0.746 |
|  | Spring Festival Effects *β_5_* | -9.08(-20.60,2.44) | 0.119 |
|  | Constant *β_0_* | 180.16(175.67,184.65) | <0.001 |
| **Osteopeptide** | |  |  |
|  | Baseline trend *β_1_* | 0.04(-0.35,0.42) | 0.842 |
|  | Change in level *β_2_* | -10.16(-30.76,10.44) | 0.322 |
|  | Change in trend *β_3_* | -0.74(-4.85,3.37) | 0.716 |
|  | COVID-19 *β_4_* | -5.57(-24.18,13.05) | 0.546 |
|  | Spring Festival Effects *β_5_* | 0.72(-15.18,16.63) | 0.926 |
|  | Constant *β_0_* | 102.66(97.74,107.58) | <0.001 |
| **Cerebral Protein Hydrolyzate** | |  |  |
|  | Baseline trend *β_1_* | -0.28(-0.48,-0.07) | 0.011 |
|  | Change in level *β_2_* | 12.28(1.45,23.11) | 0.027 |
|  | Change in trend *β_3_* | -1.68(-2.52,-0.84) | <0.001 |
|  | COVID-19 *β_4_* | 3.95(-5.08,12.99) | 0.381 |
|  | Spring Festival Effects *β_5_* | -11.77(-26.23,2.70) | 0.108 |
|  | Constant *β_0_* | 51.28(48.27,54.28) | <0.001 |
| **Vinpocetine** | |  |  |
|  | Baseline trend *β_1_* | 0.00(-0.16,0.15) | 0.984 |
|  | Change in level *β_2_* | 18.94(-5.59,43.46) | 0.126 |
|  | Change in trend *β_3_* | -2.79(-4.35,-1.23) | 0.001 |
|  | COVID-19 *β_4_* | -2.34(-21.78,17.10) | 0.809 |
|  | Spring Festival Effects *β_5_* | -9.18(-16.63,-1.74) | 0.017 |
|  | Constant *β_0_* | 90.52(88.77,92.27) | <0.001 |
| **Calf Blood Deproteinized Extract** | |  |  |
|  | Baseline trend *β_1_* | -0.18(-0.43,0.07) | 0.153 |
|  | Change in level *β_2_* | 16.63(-8.54,41.80) | 0.188 |
|  | Change in trend *β_3_* | -3.16(-6.47,0.15) | 0.061 |
|  | COVID-19 *β_4_* | 6.21(-11.17,23.60) | 0.472 |
|  | Spring Festival Effects *β_5_* | -6.62(-14.96,1.73) | 0.116 |
|  | Constant *β_0_* | 95.22(91.77,98.66) | <0.001 |
| **Cinepazide Maleate** | |  |  |
|  | Baseline trend *β_1_* | -0.18(-0.66,0.30) | 0.448 |
|  | Change in level *β_2_* | -7.26(-20.59,6.06) | 0.274 |
|  | Change in trend *β_3_* | 0.55(-0.79,1.88) | 0.409 |
|  | COVID-19 *β_4_* | 1.35(-13.86,16.56) | 0.857 |
|  | Spring Festival Effects *β_5_* | -2.66(-12.84,7.52) | 0.597 |
|  | Constant *β_0_* | 140.85(135.14,146.56) | <0.001 |
| **Guhong Injection** | |  |  |
|  | Baseline trend *β_1_* | 0.05(-0.06,0.16) | 0.398 |
|  | Change in level *β_2_* | -1.51(-4.16,1.13) | 0.251 |
|  | Change in trend *β_3_* | 0.14(-0.22,0.50) | 0.430 |
|  | COVID-19 *β_4_* | -2.25(-5.73,1.23) | 0.195 |
|  | Spring Festival Effects *β_5_* | 1.09(-1.10,3.28) | 0.317 |
|  | Constant *β_0_* | 167.99(166.70,169.29) | <0.001 |
| **Shenxiong Injection** | |  |  |
|  | Baseline trend *β_1_* | 0.52(0.45,0.60) | <0.001 |
|  | Change in level *β_2_* | 1.74(-1.97,5.45) | 0.345 |
|  | Change in trend *β_3_* | -0.44(-1.51,0.63) | 0.405 |
|  | COVID-19 *β_4_* | 0.77(-2.83,4.38) | 0.664 |
|  | Spring Festival Effects *β_5_* | 2.77(-0.35,5.88) | 0.080 |
|  | Constant *β_0_* | 68.90(68.09,69.70) | <0.001 |
| **Lugua Polypeptide Injection** | |  |  |
|  | Baseline trend *β_1_* | 0.22(-0.24,0.68) | 0.327 |
|  | Change in level *β_2_* | 2.08(-5.08,9.24) | 0.557 |
|  | Change in trend *β_3_* | 0.14(-0.45,0.72) | 0.632 |
|  | COVID-19 *β_4_* | -3.87(-8.22,0.48) | 0.079 |
|  | Spring Festival Effects *β_5_* | 4.64(-3.68,12.95) | 0.263 |
|  | Constant *β_0_* | 149.09(143.73,154.46) | <0.001 |
| **Safflower Yellow Injection** | |  |  |
|  | Baseline trend *β_1_* | -0.38(-0.69,-0.07) | 0.018 |
|  | Change in level *β_2_* | 1.52(-2.67,5.70) | 0.464 |
|  | Change in trend *β_3_* | 2.17(1.66,2.68) | <0.001 |
|  | COVID-19 *β_4_* | -18.64(-25.63,-11.66) | <0.001 |
|  | Spring Festival Effects *β_5_* | 6.52(0.77,12.27) | 0.028 |
|  | Constant *β_0_* | 106.02(101.93,110.10) | <0.001 |
